# Analysis of Fresnel Zone Plates Focusing Dependence on Operating Frequency

**DOI:** 10.3390/s17122809

**Published:** 2017-12-05

**Authors:** José Miguel Fuster, Pilar Candelas, Sergio Castiñeira-Ibáñez, Sergio Pérez-López, Constanza Rubio

**Affiliations:** 1 ETSI Telecomunicación, Universitat Politècnica de València, Camino de Vera s/n, 46022 Valencia, Spain; 2 Departamento de Comunicaciones, Universitat Politècnica de València, Camino de Vera s/n, 46022 Valencia, Spain; jfuster@dcom.upv.es; 3 Centro de Tecnologías Físicas, Universitat Politècnica de València, Camino de Vera s/n, 46022 Valencia, Spain; pcandelas@fis.upv.es (P.C.); casiser@uv.es (S.C.-I.); serpelo1@teleco.upv.es (S.P.-L.); 4 Departamento de Física Aplicada, Universitat Politècnica de València, Camino de Vera s/n, 46022 Valencia, Spain; 5 Departamento de Ingeniería Electrónica, Universitat de València, Avd. de la Universitat s/n, Burjassot, 46100 Valencia, Spain

**Keywords:** ultrasonic lens, Fresnel lens, focal depth, focal distortion, operating frequency

## Abstract

The focusing properties of Fresnel Zone Plates (FZPs) against frequency are analyzed in this work. It is shown that the FZP focal length depends almost linearly on the operating frequency. Focal depth and focal distortion are also considered, establishing a limit on the frequency span at which the operating frequency can be shifted. An underwater FZP ultrasound focusing system is demonstrated, and experimental results agree with the theoretical analysis and simulations.

## 1. Introduction

The focusing of waves has always attracted much scientific interest because of its applications in different physic areas, such as optics, microwave propagation, and acoustics [1,2,3,4,5,6,7]. To achieve wave focusing, devices based on both refraction and diffraction mechanisms can be used. Fresnel zone plates (FZPs) focus waves through constructive interference of diffracted fields [8]. They are used in situations where conventional lenses are difficult to implement [9,10]. FZPs can be classified in two main groups based on their transmission efficiency [11]. Soret FZPs are implemented alternating transparent and opaque Fresnel zones, while Rayleigh-Wood FZPs replace opaque zones with phase-reversal zones, increasing the FZP efficiency [12] by a factor of four. Ideal diffraction efficiencies for Soret and Rayleigh-Wood FZPs are 10.1% and 40.5%, respectively. The results obtained in this work are valid for both types of FZPs, although Soret FZPs have been used to demonstrate these results because of their ease of fabrication. Throughout this work, underwater acoustic transmission is considered using Soret FZPs made from brass.

Ultrasonic wave focusing has many potential applications, and different lens designs have been implemented for ultrasound and acoustic focusing. In this area, Schindel et al. [13] demonstrated the use of a micromachined Fresnel zone-plate to focus a planar ultrasonic wave in air, to a sub-millimeter spot size with a depth of field of less than 3 mm. Welter et al. [14] designed an acoustic lens with an aperiodic structure intended to operate in air using a hybrid genetic-greedy algorithm. In both cases, sound diffraction is due to large impedance mismatches between air and the lens material, and these results demonstrated the possibility of focusing ultrasound with subwavelength resolution at multiple frequencies in air using a single acoustic lens [15]. Later, Li et al. [16] reported the design of a gradient index acoustic lens by coiling up space. They proposed a model that comprises a series of acoustic metamaterial units with curled channels. Their numerical results showed that the designed acoustic metamaterial can mimic an acoustic gradient index lens with an arbitrarily large refractive index and considerably high transmission efficiency. More recently, Peng et al. [17] designed a flat sub-wavelength lens to focus acoustic waves. They analytically studied the transmission through an acoustic grating with curled slits. This grating served as a material with tunable impedance and refractive index for acoustic waves. In this work, the use of the operating frequency as a viable and dynamic control mechanism to shift the focal length in acoustic lenses for therapeutic applications is proposed.

FZPs are designed to work and focus at a design frequency. At this frequency, the behavior of the FZP is optimum and focusing at a certain focal length is achieved [18]. In most medical applications using lenses, especially in therapeutic techniques, it is critical to have a fine and dynamic control on the FZP focal length [19]. The range of focal lengths required in a certain therapeutic applications depends on the area that has to be treated. As an example, it has been reported that focal lengths ranging between 9 and 15 cm are required for the treatment of liver tumors [20].

One approach is the use of a multi-array device, which emits an ultrasonic wave with different transducers, each of them with a proper amplitude and phase so that the focus reaches the intended treatment area [21]. However, these systems are complex and expensive, and require Magnetic Resonance Imaging (MRI) real-time monitoring to adjust the amplitudes and phases of the emitting elements. Therefore, the use of focusing single element devices, such as FZPs, is a more attractive alternative, as they are cheaper and easier to use.

The effect of the operating frequency on the FZP focal length has already been reported in optics [22]. In this work, the variation of the FZP focusing parameters when working at operating frequencies different from the design frequency is analyzed for the acoustic case, and a simple focal length control mechanism is proposed for therapeutic applications. It is shown that the FZP focal length shifts almost linearly with the operating frequency, becoming a very dynamic control parameter. However, other focusing parameters, such as focal depth and focal distortion, are also affected by the operating frequency. It is shown that focal depth also depends linearly on the operating frequency in the surroundings of the design frequency, whereas focal distortion restricts the range of focal lengths available with a single FZP.

## 2. Theoretical Analysis and Simulation

In this section, the influence of the FZP operating frequency on its focusing properties is analyzed. An FZP is designed considering planar wave incidence with a focal length *F* = 10 cm, a design frequency $f_{d}=250\;\mathrm{kHz}$, and *N* = 25 Fresnel zones. With these design parameters, the Fresnel zone radii are given by [8]:

(1) $$r_{n}=\sqrt{n\lambda_{d}F+{\left(\frac{n\lambda_{d}}{2}\right)}^{2}}\;n=1,\ldots ,N$$

with $\lambda_{d}=v/f_{d}=6\;\mathrm{mm}$ being the design wavelength, and v=1500 m/s being the water sound speed.

**Figure 1:**
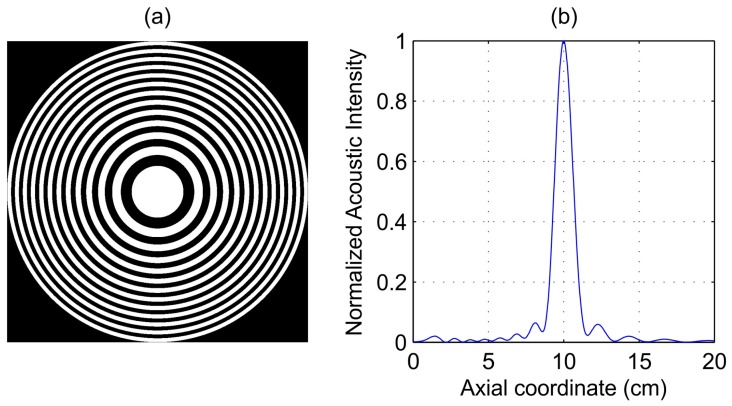
(**a**) Fresnel Zone Plate (FZP) layout; (**b**) Simulated acoustic intensity against axial distance.

Figure 1a shows the FZP layout. The white regions represent the brass opaque zones, while the black regions correspond to the transparent water-filled zones. With the design parameters stated above, the resulting FZP diameter is 28.72 cm. Figure 1b shows simulated acoustic intensity against axial distance z for an operating frequency $f_{op}=250\;\mathrm{kHz}$, identical to the design frequency. The FZP thickness is 1 mm. As it can be observed from Figure 1b, the focal length from the numerical simulation agrees with the theoretical value. The diffraction efficiency of this lens is 9.46%, which is very close to its theoretical value. 

Simulations were carried out using the Acoustic Model of COMSOL Multiphysics Modeling Software. In order to dimish the burden of the Finite Element Method (FEM), an axis-symmetric definition of the geometry was used throughout the simulations. Brass was modeled with a sound speed of $c_{b}=3600\;m/s$ and density equal to $\rho_{b}=8500\;\mathrm{kg}/m^{3}$, whereas the values used to model water were $c_{w}=1500\;m/s$ and $\rho_{w}=1000\;\mathrm{kg}/m^{3}$. To avoid reflections, a boundary condition was established at the walls of the water tank. The mesh type was set to Free Triangular, with a maximum element size of λ/8. Acoustic intensity was calculated from the acoustic pressure parameter using $I=\frac{p^{2}}{2\rho v}$, with *p* being the acoustic pressure, ρ the material density, and *v* the sound speed on the material.

**Figure 2:**
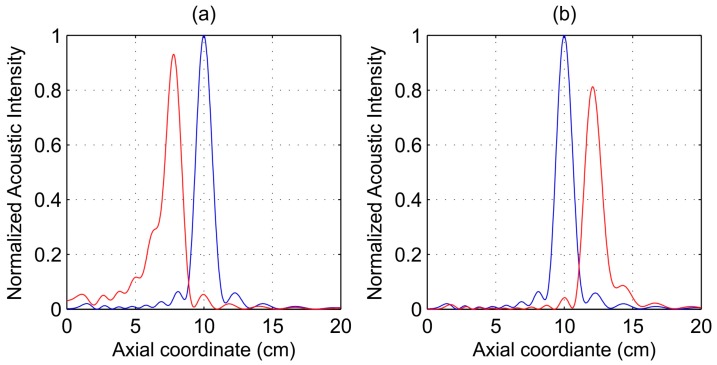
Simulated acoustic intensity against axial distance. Blue line: 250 kHz. Red line: (**a**) 220 kHz; (**b**) 280 kHz.

When the operating frequency is shifted from its design value, the FZP focal length is also modified. Figure 2a shows simulated acoustic intensity profiles against axial coordinate for operating frequencies of 220 kHz (red line) and 250 kHz (blue line). As it can be observed from Figure 2a, the FZP focal length shifted from 10.01 cm (250 kHz) to 7.80 cm (220 kHz). Figure 2b shows simulated acoustic intensity profiles when the operating frequency is shifted in the opposite direction. In this case, the focal length shifted to 12.11 cm for a 280 kHz operating frequency. The acoustic intensity profiles were normalized to the optimum case, that is when the FZP is working at the operating frequency of 250 kHz. As it can be observed from Figure 2, when the operating frequency is modified and the focal length shifts from its design value, a slight decrease in the peak intensity is appreciated in both directions, and it is more significant when the focal length is increased.

The focal length at an operating frequency different from the design frequency can also be theoretically obtained using Equation (2):

(2) $$F\left(f_{op}\right)=\frac{R^{2}}{Nv}f_{op}-\frac{Nv}{4f_{op}}$$

with $f_{op}$ being the operating frequency, *R* the FZP external radius, *N* the number of Fresnel zones, and *v* the water sound speed.

**Figure 3:**
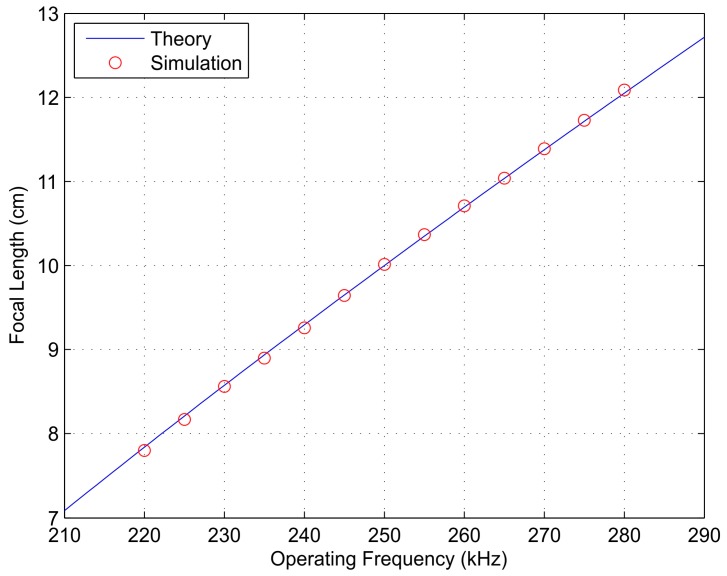
Focal length against operating frequency: theory (blue solid line) and simulation (red dots).

The theoretical focal lengths corresponding to the frequencies considered in Figure 2, 220, 250, and 280 kHz, are 7.84, 10.00, and 12.09 cm, respectively, which shows an excellent agreement between theoretical and simulation results. Additionally, Figure 3 depicts the FZP focal length as a function of the operating frequency. The blue solid line is obtained theoretically from the direct application of Equation (2), while red dots correspond to simulation results for operating frequencies ranging from 220 to 280 kHz with a 5-kHz step. From Equation (2), it can be concluded that the focal length variation against the operation frequency is not a completely linear dependence, although it becomes very close, as shown Figure 3. This is due to the fact that the first term on Equation (2) is dominant for the range of operating frequencies currently considered. The weight of this second term may vary when some of the design parameters, such as the frequency range, the number of Fresnel zones, or the FZP external radius, are modified. In general, Equation (2) becomes more linear when either the frequency or the FZP external radius are augmented or when the number of Fresnel zones is diminished. However, Equation (2) can be regarded as an almost linear relation for the range of frequencies used in ultrasonics and typical values for *R* and *N*.

Additional focusing parameters, such as the focal depth and the focal distortion $D_{f}$, can also be analyzed from simulation. The focal depth is estimated through the full width half maximum (FWHM) of the focusing profile, which corresponds to the distance between the two points adjacent to the maximum value at which the normalized acoustic intensity reaches half its maximum value.

**Figure 4:**
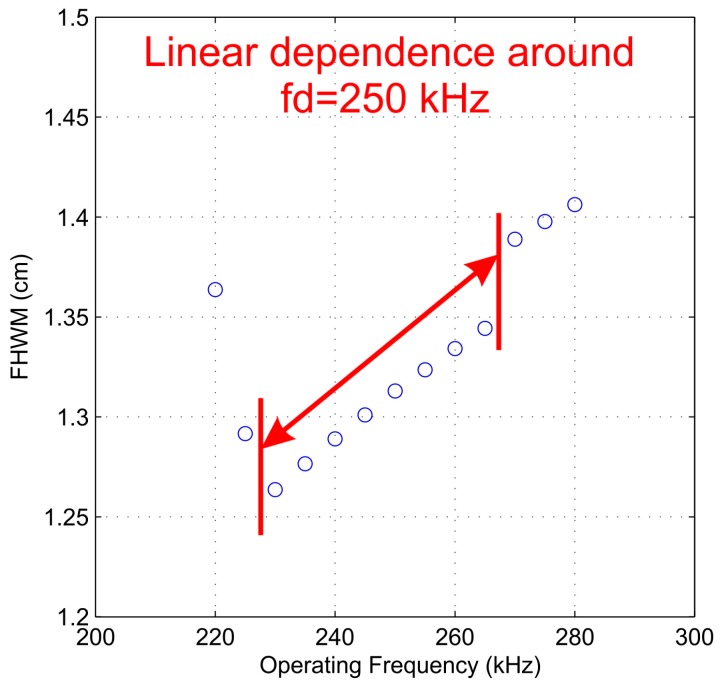
Full Width Half Maximum (FHWM) against operating frequency (simulation results).

Figure 4 shows the FWHM simulation results for the same range of operating frequencies considered in Figure 3, ranging from 220 to 280 kHz with a 5-kHz step. As it can be observed from Figure 4, when the operating frequency is close to the design frequency, there is a linear relation between the FWHM and the operating frequency. However, when the operating frequency is shifted to further values, the linear relation no longer stands.

The FZP focal distortion $D_{f}$ was estimated from the variations between the focus profiles of two different FZPs of the same size at a particular operating frequency. One FZP is the current FZP being analyzed, while the reference FZP has a design frequency equal to the operating frequency. This parameter was mathematically defined using the Normalized Mean Square Error (NMSE) between both focusing profiles [23] as:

(3) $$D_{f}\left(f_{op}\right)=\frac{\sum_{z}{\left(I_{f_{op}}-I_{fd}\right)}^{2}}{\sum_{z}{\left(I_{f_{op}}\right)}^{2}}$$

with $I_{f_{op}}$ being the acoustic intensity for an FZP designed and operating at the operating frequency, and $I_{fd}$ being the acoustic intensity for an FZP designed for the designed frequency (250 kHz) and operating at the operating frequency.

**Figure 5:**
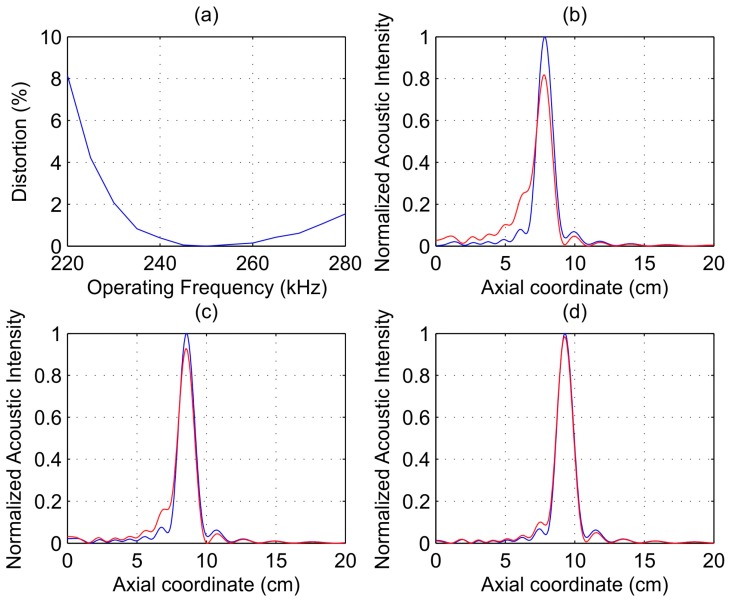
(**a**) Focal distortion against operating frequency; (**b**–**d**) Simulated acoustic intensity against axial distance for an FZP designed at the operating frequency (blue solid line) and for an FZP designed at 250 kHz (red solid line): (**b**) $f_{op}$ = 220 kHz; (**c**) $f_{op}$ = 230 kHz; and (**d**) $f_{op}$ = 240 kHz.

Figure 5a shows focal distortion against the operating frequency. As it can be observed from Figure 5a, focal distortion presents a smooth behavior and it is minimal when the operating frequency is equal to its design counterpart. Figure 5b–d show focusing profiles for different operating frequencies: 220, 230, and 240 kHz, respectively. The blue solid line corresponds to the acoustic intensity of the reference FZP, while the red solid refers to the current FZP being analyzed. The acoustic intensity profiles were normalized to the reference case, that is when the FZP is operating at the design frequency. As it can be observed from Figure 5, the difference between both focusing profiles is more significant when the operating frequency is shifted further from its design value. The intensity profile changes its distribution along the axial coordinate when distortion increases, lowering its maximum value and widening around the focus position. If a maximum focal distortion is established, the range of operating frequencies is then effectively reduced. As an example, an admitted 1% maximum focus distortion limits the current operating frequency range to 41 kHz and the focal length range to 3 cm. The maximum admitted distortion will depend on the requirements of the specific application, but in most cases distortions below 2% would assure a good overall performance.

## 3. Experimental Results and Discussion

**Figure 6:**
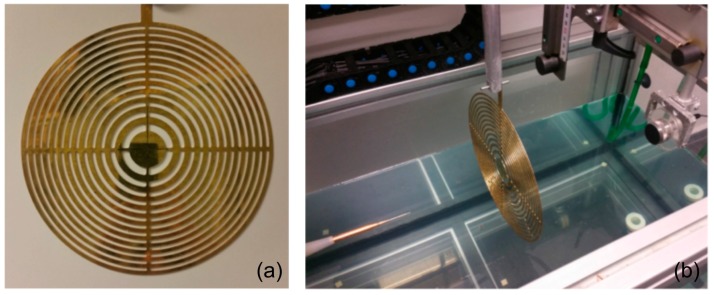
(**a**) Brass Soret-type FZP layout; (**b**) Experimental setup.

The experimental setup uses the ultrasonic immersion transmission technique. The assembly is placed in a water tank. A piston transducer from Imasonic, with a 250 kHz center frequency and an active diameter of 32 mm, is employed as emitter and a needle hydrophone, from Precision Acoustics Ltd., is employed as receiver. This hydrophone has a diameter of 1.5 mm and a ±4 dB bandwidth span from 200 kHz to 15 MHz. An automated positioning system built around the water tank is used to align and position the hydrophone at a three-dimensional (3D) grid of measurement points located inside the tank. The brass FZP shown in Figure 6a is used to conform the focus profile. *F* = 5 cm, $f_{d}$ = 250 kHz, and *N* = 27 Fresnel zones were selected as the FZP design parameters for this setup. The FZP thickness is 1 mm. The distance between the piston emitter and the brass FZP is 35 cm. The acoustic signal launched by the emitter is detected by the hydrophone, acquired and digitalized using a digital PC oscilloscope (Pico Technology, St Neots, UK). Time domain data are averaged and scanning is performed with the automated positioning system along a plane normal to the FZP, with a spatial resolution of 1 × 1 mm^2^. The experimental setup is shown in Figure 6b.

**Figure 7:**
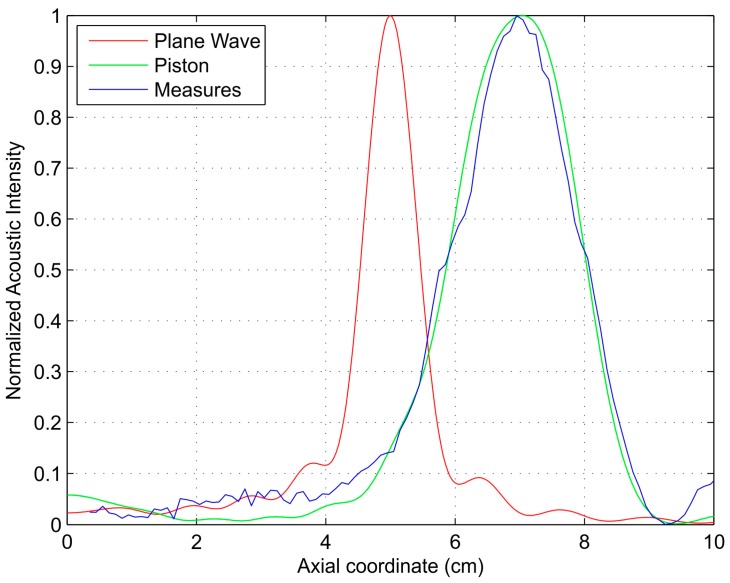
Normalized axial acoustic intensity against axial coordinate: simulated plane wave incidence model (red line), simulated piston model (green line), experimental measurements (blue line).

As mentioned above, a piston transducer is used as the ultrasound transmitter. The piston transducer produces a wave front that introduces a phase error compared to the plane wave due to the path difference. This path difference varies between 0 and 0.07 λ at the center of the FZP and between 2.73 λ and 4.77 λ at the outer FZP locations. In the rest of the FZP plate, the path difference is somewhere in between. Therefore, there is no real plane wave excitation, and the focus profile is affected through a shift on the focus location from the expected design value. Figure 7 shows normalized acoustic intensity profiles against axial position for a 250 kHz operating frequency. These profiles correspond to simulated plane wave excitation (red line), simulated piston excitation (green line), and experimental piston excitation (blue line).

At an operating frequency of 250 kHz, the use of a piston transducer as an emitter instead of plane wave excitation translates into a shift on the focal length from 5.0 cm (design value) to 6.9 cm. The experimental focal length is 7.1 cm, which agrees very well with the simulation results.

**Figure 8:**
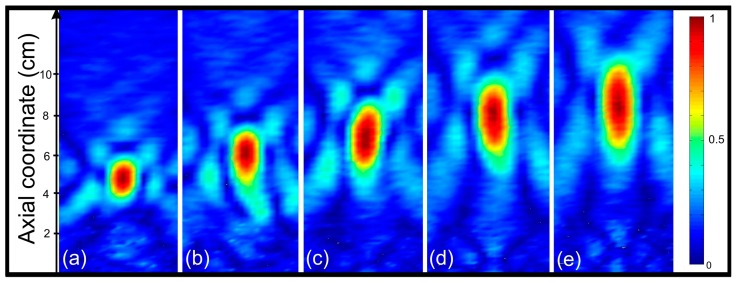
Measured normalized acoustic intensity maps for different operating frequencies: (**a**) 210 kHz; (**b**) 230 kHz; (**c**) 250 kHz; (**d**) 270 kHz; (**e**) 290 kHz.

Figure 8 shows the normalized measured intensity maps in the transmission direction for a 35-cm piston-FZP separation and different operation frequencies ranging from 210 to 290 kHz with a 20-kHz step. As it can be observed from Figure 8, the focus location varies its position when the operating frequency is modified. The focal length increases with the operating frequency, as expected from the theoretical analysis.

**Figure 9:**
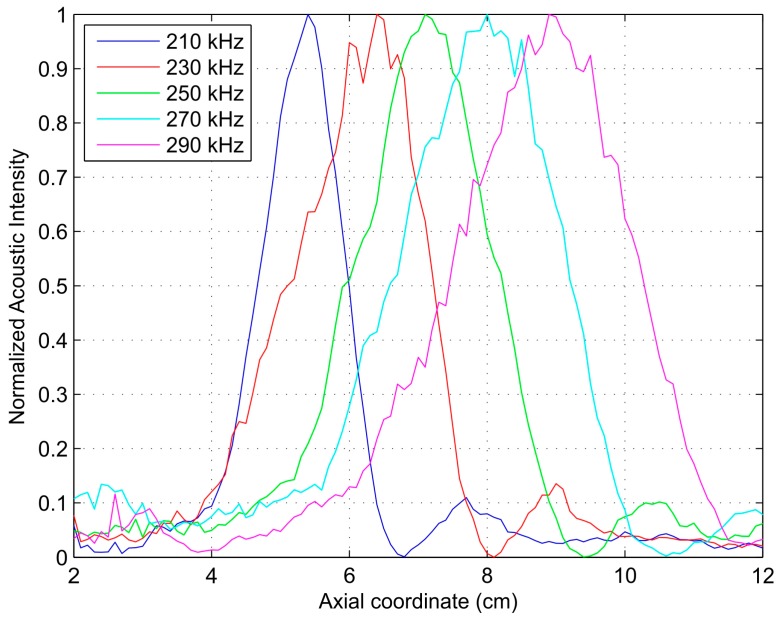
Measured normalized axial acoustic intensity against axial coordinate.

Figure 9 depicts the measured normalized acoustic intensity profiles against the axial coordinate for the previous five different operating frequencies: 210, 230, 250, 270, and 290 kHz. The corresponding focal lengths for these operating frequencies are 5.4, 6.4, 7.1, 8.0, and 8.9 cm, respectively.

**Figure 10:**
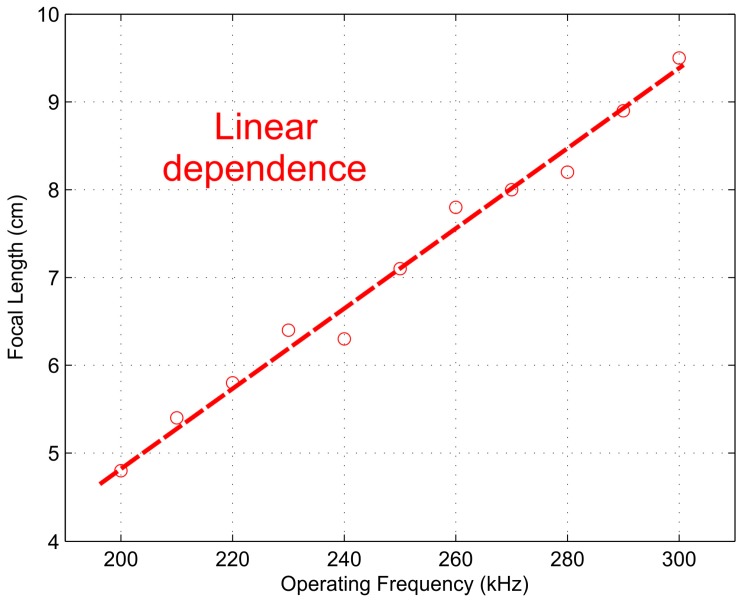
Measured focal length against operating frequency.

**Figure 11:**
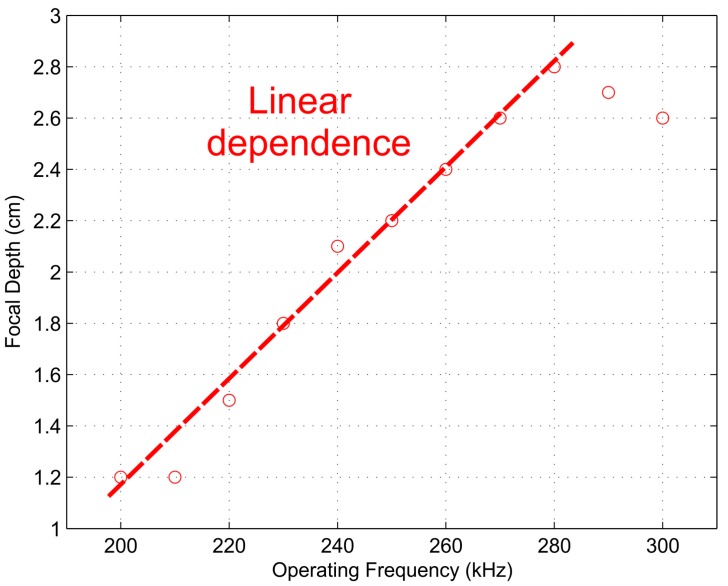
Measured focal depth against operating frequency.

Figure 10 and Figure 11 depict the measured FZP focal length and focal depth, respectively, against the operating frequency. Although a piston emitter was used in the experimental setup, the linear relations stated previously in the simulation results still can be observed from these figures. Figure 10 shows a linear relation in the whole frequency span, while Figure 11 shows that the linear relation is held for a wide frequency range around the design frequency, between 210 and 280 kHz. Therefore, an effective and dynamic method to modify the FZP focal length was achieved.

## 4. Conclusions

A thorough analysis of the dependence of focusing profiles on the operating frequency has been carried out for FZPs. Simulations and experimental measurements do agree, and confirm that there is an almost linear dependence between the FZP focal length and the operating frequency, allowing a fine dynamic control mechanism that can be helpful in many different applications that use ultrasound focusing, such as medical therapeutic treatments. It has been also shown that the focal distortion limits the range of operating frequencies available in a particular application. The results of this study have been demonstrated for underwater acoustic transmission using a brass Soret FZP.

## References

[1] Goodman J.W. (2016). Introduction to Fourier Optics.

[2] Marchand E.W. (1978). Gradient Index Optics.

[3] Stamnes J.J. (1986). Waves in Focal Regions: Propagation, Diffraction and Focusing of Light, Sound and Water Waves.

[4] Minin I.V., Minin O.V. (2011). Reference Phase in Diffractive Lens Antennas: A Review. J. Infrared Millim. Terahertz Waves.

[5] Gomez-Lozano V., Candelas P., Belmar F., Rubio C., Uris A. (2014). Ultrasonic lens based on a subwavelength slit surrounded by grooves. Sensors.

[6] Li Y., Yu G., Liang B., Zou X., Li G., Cheng S., Cheng J. (2014). Three-dimensional Ultrathin Planar Lenses by Acoustic Metamaterials. Sci. Rep..

[7] Rubio C., Fuster J.M., Castiñeira-Ibáñez S., Uris A., Belmar F., Candelas P. (2017). Pinhole Zone Plate Lens for Ultrasound Focusing. Sensors.

[8] Cao Q., Jahns J. (2004). Comprehensive focusing analysis of various Fresnel zone plates. J. Opt. Soc. Am. A.

[9] O’Shea D.C., Suleski T.J., Kathman A.D., Prather D.W. (2003). Diffractive Optics: Design, Fabrication, and Test.

[10] Zhang S., Yin L., Fang N. (2009). Focusing Ultrasound with an Acoustic Metamaterial Network. Phys. Rev. Lett..

[11] Hristov D. (2000). Fresnel Zones in Wireless Links, Zone Plate Lenses and Antennas.

[12] Calvo D.C., Thangawng A.L., Nicholas M., Layman C.N. (2015). Thin Fresnel zone plate lenses for focusing underwater sound. Appl. Phys. Lett..

[13] Schindel D., Bashford A., Hutchins D. (1997). Focusing of ultrasonic waves in air using a micromachined Fresnel zone-plate. Ultrasonics.

[14] Welter J.T., Sathish S., Christensen D.E., Brodrick P.G., Heebl J.D., Cherry M.R. (2011). Focusing of longitudinal ultrasonic waves in air with an aperiodic at lens. J. Acoust. Soc. Am..

[15] Welter J.T., Sathish S., Dierken J.M., Brodrick P.G., Cherry M.R., Heebl J.D. (2012). Broadband aperiodic air coupled ultrasonic lens. Appl. Phys. Lett..

[16] Li Y., Liang B., Tao X., Zhu X., Zou X., Cheng J. (2012). Acoustic focusing by coiling up space. Appl. Phys. Lett..

[17] Peng P., Xiao B., Wu Y. (2014). Flat acoustic lens by acoustic grating with curled slits. Phys. Lett. A.

[18] Gómez Álvarez-Arenas T.E., Camacho J., Fritsch C. (2016). Passive focusing techniques for piezoelectric air-coupled ultrasonic transducers. Ultrasonics.

[19] Ter Haar G., Coussios C. (2007). High intensity focused ultrasound: Physical principles and devices. Int. J. Hyperth..

[20] Kennedy J.E., Wu F., Ter Haar G.R., Gleeson F.V., Phillips R.R., Middleton M.R., Cranston D. (2004). High-intensity focused ultrasound for the treatment of liver tumors. Ultrasonics.

[21] Carovac A., Smajlovic F., Junuzovic D. (2011). Application of Ultrasound in Medicine. Acta Inform. Med..

[22] Markovich H., Filonov D., Shishkin I., Ginzburg P. Bifocal Fresnel Lens Based on the Polarization-sensitive metasurface. http://arxiv.org/pdf/1707.03652.

[23] Gollapudi S. (2016). Practical Machine Learning.

